# Routine Multimodal Antiemesis Including Low-Dose Perphenazine in an Ambulatory Surgery Unit of a University Hospital: A 10-Year History

**DOI:** 10.1100/tsw.2007.132

**Published:** 2007-06-12

**Authors:** Brian A. Williams, Michael L. Kentor, Susan J. Skledar, Steven L. Orebaugh, Manuel C. Vallejo

**Affiliations:** ^1^ University of Pittsburgh Medical Center, Pittsburgh, PA, USA; ^2^ Department of Anesthesiology, University of Pittsburgh School of Medicine, Pittsburgh, PA, USA; ^3^ University of Pittsburgh School of Pharmacy and Director - Drug Use and Disease State Management Program, University of Pittsburgh Medical Center, Pittsburgh, PA, USA

**Keywords:** postoperative nausea and vomiting, PONV, prophylaxis, multimodal prophylaxis, perphenazine, cyclizine, aprepitant, 5-HT_3_ antagonists, emesis

## Abstract

For 10 years, we have used intravenous and oral perphenazine as part of a multimodal antiemetic prophylaxis care plan for at least 10,000 outpatients. We have never encountered an adverse event, to our knowledge, when the intravenous dose was less than or equal to 2 mg, or when the single preoperative oral dose did not exceed 8 mg (with no repeated dosing). As a single-dose component of multimodal antiemetic prophylaxis therapy, we believe that this track record of anecdotal safety in adults who meet certain criteria (age 14–70, no less than 45 kg, no history of extrapyramidal reactions or of Parkinson disease, and no Class III antidysrhythmic coadministered for coexisting disease) constitutes a sufficient patient safety basis for formal prospective study. We believe that future perphenazine studies should include routine coadministration with prospectively established multimodal antiemetics (i.e., dexamethasone and a 5-HT_3_ antagonist). In settings where droperidol is still routinely used and deemed acceptable by local scientific ethics committees, we believe that oral perphenazine 8 mg should be compared head to head with droperidol 0.625–1.25 mg in patients receiving coadministered dexamethasone and 5-HT_3_ antagonists in order to determine differences in synergistic efficacy, if any. Similar trials should be performed, individually evaluating cyclizine, transdermal scopolamine, and aprepitant in combination with coadministered dexamethasone and a 5-HT_3_ antagonist. Such studies should also quantify efficacy in preventing nausea and vomiting after discharge home, and also quantify the extent to which the prophylaxis plans reduce postanesthesia care unit (PACU) requirements (i.e., increase PACU bypass), reduce the need for any nursing interventions for postoperative nausea and/or vomiting (PONV), and influence the extent to which any variable costs of postoperative nursing care are reduced.

## INTRODUCTION

Recent consensus guidelines for the prevention of postoperative nausea and/or vomiting (PONV)[1] do not call for the prophylaxis of PONV in "low-risk" patients. One or two interventions are suggested for patients at "medium risk", based on a circulated draft of revised recommendations from the Society for Ambulatory Anesthesia (accessed November 3, 2006). Since these consensus statements, research has indicated that the incremental addition of mechanistically unique, antiemetic drugs reduces PONV risk by 22–26% (when studying ondansetron, droperidol, and dexamethasone)[2]. This important latter finding, in our opinion, calls for the reconsideration of the former recommendation.

It is difficult to quantify the extent to which routine droperidol use is withheld due to concerns about its Black Box listing by the Food and Drug Administration (FDA), based on there being no guarantee of medicolegal protection with its use. Additionally, prophylaxis with ondansetron and dexamethasone appears unlikely to protect against dopaminergic receptor-mediated PONV. Therefore, full risk reduction with the antidopaminergic mechanism suggested by current consensus guidelines may never be realized, and patients in certain settings may suffer. Conversely, practitioners choosing to conservatively monitor patients for hours after droperidol dosing may create unwanted patient care congestion, with a potentially significant variable cost if forced overtime or unplanned hospital admissions are incurred as a result.

Additional research is needed to determine if other antidopaminergics (e.g., perphenazine) can be the therapeutic equivalent of droperidol when calculating cumulative risk reduction (if not possibly of better quality due to possible side effect risk reduction)[3].

Other antiemetic mechanisms (e.g., antihistamine, anticholinergic, neurokinin-1 antagonist) also require further study with respect to cumulative risk reduction. To our knowledge, there is no evidence indicating that any individual mechanism is a precursor, or would preempt, another mechanism.

We particularly mention perphenazine, since we (authors BAW and MLK) have used this medication for 10 years in over 10,000 patients, with no patient complications when the later-described dosing thresholds were not exceeded. In this report, we will summarize our previously published (retrospective) clinical experience with perphenazine in combination with other antiemetic agents in our group's efforts toward "zero tolerance" for PONV. We will then provide simple calculations of risk reductions, assuming: (i) perphenazine proves to be equivalent to droperidol; and (ii) triple-agent antiemesis (with perphenazine, dexamethasone, and ondansetron) are used in patients with PONV risks of 10% or higher.

## REVIEW OF OUR INSTITUTION'S EXPERIENCE WITH PERPHENAZINE IN COMBINATION WITH OTHER ANTIEMETICS IN OUTPATIENT ORTHOPEDIC SURGERY, AND CALL FOR EXPANDING THE RESEARCH AGENDA FOR ORAL ANTIEMETIC PROPHYLAXIS

### A Brief History of Perphenazine as a Perioperative Antiemetic

To our knowledge, the first detailed description of perphenazine (in comparison with several other first-generation tranquilizers) as a perioperative sedative/antiemetic was in the 1960s by Dundee et al. In one report, several opioid-tranquilizer strategies were compared, showing perphenazine to be not a particularly good premedicant due to its relative lack of sedative effects[4]. In this report, perphenazine-treated patients were less likely to encounter PONV, and this finding led to two other studies by the same author's research group. Perphenazine at 5 mg i.m. (as monotherapy) proved to be more efficacious (against opioid-induced emesis) than perphenazine 2.5 mg, and was equiefficacious with cyclizine 50 mg i.m.[5]. This report also commented that perphenazine 5 mg i.m. produced an unacceptably high incidence of restlessness, compared with cyclizine 50 mg i.m. A decade later, the same group verified the higher efficacy of cyclizine 50 mg i.m. over perphenazine 2.5 mg i.m.[6].

In the mid-1970s, oral perphenazine showed 20% bioavailability[7]. We are not aware of any additional bioavailability studies since then. We are not aware of any studies/case reports of perphenazine-induced ECG changes in the perioperative setting with single doses. Perphenazine (in a study of schizophrenic patients receiving an oral dose of 20 mg/day, with or without coadministered amitriptylene, for 12 weeks) appeared to have associated electrocardiogram (ECG) abnormalities after glucose loading (but not fasting)[8]. In one case report from 1977, a 45-year-old woman who was concomitantly hypokalemic and being chronically treated with combined perphenazine-amitriptyline developed Q-T prolongation[9].

In 1995, Desilva et al. showed perphenazine to be more effective than placebo, with fewer side effects than droperidol (see below)[3].

In 1997, Splinter et al. described how perphenazine was more efficacious than placebo for any vomiting after tonsillectomy in children[10]. When comparing perphenazine with dexamethasone in a similar study, this group found the former more effective in hospital, with no difference after discharge home[11]. In children undergoing tonsillectomy, Fujii et al. (1999) described that there were no significant differences in vomiting during the first 3 h after anesthesia when comparing granisetron 40 mcg/kg i.v. vs. perphenazine 70 mcg/kg i.v.; however, sustaining vomiting prophylaxis was more successful with granisetron vs. perphenazine[12]. A repeat study by the same authors using oral granisetron 40 mcg/kg (which shows 60% bioavailability) vs. oral perphenazine 70 mcg/kg (which shows 20% bioavailability) showed that granisetron was more efficacious than perphenazine against postoperative vomiting throughout the first 24 h[13]. Given the inequalities of the bioavailability-based dose adjustment, this finding is hardly surprising. Perphenazine is available as an option for pediatric PONV according to consensus guidelines[1].

## Institutional Experience with Perphenazine

We (authors BAW and MLK) began using perphenazine in 1997, soon after reviewing the report of Desilva et al. that described fewer side effects with perphenazine than with monotherapies consisting of droperidol, metoclopramide, or ondansetron[3]. Unlike the perphenazine 5 mg i.v. dose used in this study, we combined perphenazine 1.25–2.0 mg i.v. with dexamethasone 4–10 mg i.v., based on abstract presentations regarding dexamethasone at scientific meetings that evolved into subsequent publications[14,15].

Our rationale for adding routine multimodal antiemetic prophylaxis at this point was to help reduce PONV in our institution, which reached 39% in an index population of patients having undergone anterior cruciate ligament (ACL) reconstruction with general anesthesia (and discretionary antiemetic prophylaxis with zero or one agent)[16]. Due to acquisition cost concerns, ondansetron and the serotonin receptor type 3 (5-$HT_3$) antagonists were relegated by our pharmacy and therapeutics committee to "rescue only after other antiemetic rescue agents have failed." In our general anesthesia (volatile agent) clinical pathway for outpatient orthopedics[17], the addition of two antiemetics trended toward a lower PONV rate of 25% (66/267) when compared with 33% (92/276) in patients receiving one or fewer antiemetics ($p = 0.07$). Perphenazine and dexamethasone were used in the majority of cases where two antiemetics were used (based on authors' BAW and MLK practice patterns vs. overall caseload sampled from the group practice), while droperidol was the sole antiemetic in most cases when one antiemetic was used in this retrospective review (based on group practice patterns at the time). We found no risk reduction of using one antiemetic in this clinical pathway vs. no antiemetics. This may have been due to the lack of efficacy of droperidol in men[2], who made up 63% of our patients in our outpatient orthopedic dataset[17].

More dramatic was the PONV risk reduction seen in our regional anesthesia (RA) clinical pathway, in which 20% of RA patients encountered PONV when one or fewer antiemetics were given vs. 9% when two antiemetics were used (again perphenazine and dexamethasone 90% of the time, $p < 0.001$)[17].

From the same review, we noted an associated 36% risk reduction in postdischarge nausea and vomiting (PDNV)(n = 614 patients retrospectively analyzed, $p = 0.024$) when the perphenazine-dexamethasone combination was used vs. those in whom the combination was not used[17]. The use of volatile agents was not predictive of PDNV in this retrospective survey.

We have published data specific to patients having undergone ACL reconstruction in addition to the 39% rate after general anesthesia (n = 50/127)[16]. When RA was used for ACL reconstruction, the PONV rate was 18% (n = 64/347); perphenazine and dexamethasone (but not ondansetron with or without dexamethasone) was associated with less PONV (odds ratio = 0.3, $p = 0.005$)[18].

When we learned that dexamethasone and ondansetron in combination was associated with less PDNV (when compared with ondansetron alone or droperidol)[19], we then combined perphenazine, dexamethasone, and ondansetron together as our group practice's antiemetic prophylaxis for outpatient orthopedic surgery. These retrospective findings, along with the findings of Gupta et al.[19] regarding postdischarge benefits of ondansetron-dexamethasone (but not droperidol), are sufficiently compelling to justify formal study of all three agents (perphenazine-dexamethasone-ondansetron) for routine multimodal antiemetic prophylaxis to prevent PDNV.

In a prospectively studied RA patient sample undergoing ACL reconstruction, this three-agent antiemetic combination was associated with a low 4% PONV rate on the day of surgery[20]. Interestingly, these patients received intra-articular neostigmine and meperidine, as well as low-dose intravenous ketamine[20], all of which are typically considered emetogenic once specific systemic thresholds are reached. Nausea, vomiting, or retching were reported more frequently than "some of the time" in no more than 6% of patients on any of the first 4 postoperative days[21]. In the context of the described standardized antiemetic technique with RA, the best predictor of avoiding nausea-vomiting-retching after discharge home was avoiding any previous nausea-vomiting-retching on the day of surgery or on any day after surgery[21]. This finding in RA patients verified the same finding in general anesthesia patients reported 12 years previously[22]. Therefore, "antiemetic momentum" may prove to be extremely important for patient outcomes, which in our opinion contradicts the "wait and see" consensus guideline for "low-risk" patients with a predicted 10–20% PONV incidence if left untreated.

In our orthopedic outpatient clinical pathway from 2002 to 2006, patients were to receive preoperative oral perphenazine 8 mg plus ondansetron 4 mg i.v. intraoperatively. In this time period, there were 6605 patients who were within the parameters of described clinical pathway and 2853 patients who were not within pathway parameters (i.e., no preoperative perphenazine was given in these 2853 patients). In this clinical pathway, a repeat dose of ondansetron 4 mg i.v. in the postanesthesia care unit (PACU) was the first-choice antiemetic rescue medication. The comparative ondansetron rescue rates, based on perphenazine prophylaxis, were 12.8% (with perphenazine) and 17.6% (without perphenazine). Therefore, in this large sample clinical pathway, we observed an associated 27.2% reduction in the rate of ondansetron rescue in orthopedic patients who received perphenazine pretreatment, providing compelling retrospective data justifying rigorous prospective study.

To summarize our 10-year experience, we have published reports regarding reduced PONV from approximately 40 to 4% in ACL reconstruction in our institution by administering the described multimodal antiemetics. This risk reduction took place without any formal accounting for the usual generally accepted risk factors. We believe that we have reasonable retrospective evidence that the combination of perphenazine, dexamethasone, and ondansetron may not only have PONV benefits, but also potentially sustained antiemetic benefits after discharge. We believe that our specialty's current consensus statement should call for more detailed prospective analysis of oral perphenazine in the routine multimodal context. Perphenazine does not have a "Black Box" warning from the FDA.

## Future Research Considerations

We have very limited clinical experience with cyclizine (the off-patent antihistamine), but the preferable lower esophageal sphincter activity of cyclizine vs. droperidol[23], and cyclizine's therapeutic equivalence with droperidol in rare studies[24], should call for additional prospective study or at least detailed retrospective clinical review of cyclizine use in hospital datasets. Cyclizine is preferable to dimenhydrinate in the aerospace medicine literature (relevant to motion sickness)[25]. Would oral cyclizine also provide a 25% cumulative risk reduction via separate antihistamine activity as part of a preoperative multimodal antiemetic strategy? Studies are needed to determine its potential value in risk reduction, perhaps in comparison with perphenazine, or possibly in comparison with (or combined with) patented oral antiemetics, such as the substance-P antagonist aprepitant.

In a very brief clinical anecdotal experience, we found that the preoperative combination of oral perphenazine 4–8 mg and oral cyclizine 50 mg was associated with significant postoperative sedation, which led to unwanted PACU admissions based on our published PACU bypass criteria[26]. That said, we have not seen postoperative sedation as problematic with perphenazine when given (without cyclizine) intravenously (2.0 mg or less) or orally (8 mg or less preoperatively), in the context of high-volume PACU bypass using documented criteria[26]. Likewise, we have never seen extrapyramidal reactions in adolescent or adult patients (up to 70 years old) with these perphenazine doses, assuming that they are not given to patients with such reactions to phenothazines, or to patients with Parkinson disease.

Another factor warranting additional research consideration is the notion of whether nursing labor costs for PONV rescue is a fixed vs. variable cost. Specifically, at what capacity threshold for a hospital or ambulatory surgery facility, and at what PONV incidence level, does PONV rescue become a variable cost for nurse staffing as opposed to a fixed cost? Intuitively, if (1) all PONV is treated on a rescue basis and all patients are successfully discharged home, (2) no staff incur forced overtime (and/or additional staffing is not needed), and (3) PACU congestion does not forbid operating room exit as soon as surgery is complete, then all staffing costs are going to be fixed costs. We are not aware of any facilities in our multihospital system that meet all of these assumptions associated with relative overstaffing and/or unfilled capacity.

With respect to the potential for routine multimodal antiemesis to reduce "phase 2" nursing workload after PACU bypass, we report on the following anecdote. Outpatient shoulder surgery in our institution is routinely performed under interscalene nerve block and propofol infusion, and no volatile agents or airway devices. We previously reported that this care plan was associated with a 9% PONV rate (13/146) when perphenazine and dexamethasone were used as multimodal antiemetics, and a 16% (50/303, $p < 0.005$) PONV rate when one or fewer antiemetics were used[17]. After additional query of our original database of these 449 shoulder surgery outpatients, we found that PACU bypass rate was 94%. There was no associated increase in the need for any "phase 2" nursing interventions after PACU bypass, but this finding is likely underpowered due to the low incidence of PACU admission. However, antiemetic prophylaxis with perphenazine and dexamethasone in these patients was associated with a reduced need for a "phase 2" nursing intervention by 50%. Hospital discharge times were 162 min (151, 173, 95% confidence interval) in patients receiving perphenazine-dexamethasone vs. 178 min (169, 186; $p = 0.034$) in patients who did not receive perphenazine-dexamethasone[27]. These anecdotes from our quality-control database should be interpreted solely as reasonable justification for continued hypothesis testing regarding the role of multimodal antiemetic prophylaxis in an effort to achieve both PACU bypass as well as documenting no additional nursing workload increases after PACU bypass. Even though PACU bypass is a phenomenon largely concentrated in North America, any antiemetic option that does not prolong PACU stay should be of some international consideration at least.

## Considerations Regarding Potential for Harm

With regard to comparative side effects of droperidol 1.25 mg i.v. vs. perphenazine 5 mg i.v., Desilva et al.[3] described no restlessness in 57 patients receiving perphenazine, but 4/55 receiving droperidol ($p$ <

0.05). In addition, one perphenazine patient (2%) encountered sedation vs. 12 droperidol patients (22%, $p < 0.05$)[3]. Certainly, more comparative studies are needed, especially evaluating lower doses of perphenazine in a multimodal dosing scheme. One could presume that lower perphenazine doses should lead to even fewer side effects.

With respect to ondansetron, it is known that 3% of treated patients encounter headache attributable to ondansetron[28], while droperidol appears to protect somewhat against headache after general anesthesia (number needed to treat = 25)[29]. Whether perphenazine may be similarly protective against headache (anesthesia induced or ondansetron induced) requires further study. In our practice, we routinely administer acetaminophen (and celecoxib if not sulfa-allergic) preoperatively for postoperative analgesia. It seems conceivable that these analgesics may attenuate the occurrence of headache in our clinical practice of routine ondansetron administration. Other potential harms that are statistically significant include elevated liver enzymes and constipation[30].

Dexamethasone given as a single dose is not associated with adverse events[31], and its use as an analgesic adjunct has recently been supported in a leading orthopedic journal[32].

## RECALCULATING RISK WITH ROUTINE PROPHYLAXIS USING PERPHENAZINE, DEXAMETHASONE, AND ONDANSETRON FOR PATIENTS WITH 10% RISK OR GREATER

Apfel et al.[2] nicely defined the estimated incidence of PONV as a function of baseline risk. Volatile agent use carries a baseline 10% risk on which additive risks related to gender (female), past history of PONV/motion sickness, opioid use, and nonsmoking status bring additional risk increments for patients undergoing general anesthesia[2]. These risk factors have not been verified in patients receiving RA. This section will consider the potential risk reduction associated with multimodal antiemesis (specifically perphenazine-dexamethasone-ondansetron). The primary assumption is that oral perphenazine would prove to be equiefficacious as intravenous droperidol, but with no side effects in the low doses described (based on our 10-year clinical experience).

"Low-risk" patients (zero or one, and/or receiving a volatile agent) treated with the "wait and see" algorithm would encounter up to a 20% PONV rate. If there were no contraindications to perphenazine-dexamethasone-ondansetron, then the estimated incidence would be 4–8% (Table 1).

Patients with "moderate risk" (assumes two risk factors plus general anesthesia, leading to a 40% risk without prophylaxis) are recommended "one or two" antiemetics for prophylaxis (e.g., droperidol plus dexamethasone, droperidol plus ondansetron, or ondansetron plus dexamethasone) according to consensus guidelines. This would lead to a 22–29% incidence of PONV. The same "moderate" risk category treated with perphenazine-dexamethasone-ondansetron would encounter a 16% incidence of PONV (Table 1).

"High-risk" patients (assuming three to four risk factors and a 60–80% risk if not given prophylaxis) are recommended two or more interventions plus a multimodal approach incorporating as many techniques as possible including RA and total intravenous anesthesia. When considering only the two to three available recommended prophylactic antiemetics (from droperidol, ondansetron, and dexamethasone) before other multimodalities, this would reduce the estimated incidence to 24–44%. By providing high-risk patients perphenazine-dexamethasone-ondansetron routinely, the estimated incidence would range from 24–32% before the opportunity for other antiemetic multimodalities were applied (Table 1).

Table 1 summarizes the number of patients that would be adversely affected with PONV by following the consensus statement, in comparison with the 32% risk reduction that appears possible via the perphenazine-dexamethasone-ondansetron technique, based on our group's evolving years of success with perphenazine as our choice antidopaminergic. Perphenazine does not seem to carry any regulatory-related apprehensions ("Black Box" warnings; recommended or required 2-h recovery room stays for ECG monitoring), or any apparent side effects in the dose ranges listed.

**TABLE 1**
**Side-by-Side Estimated Incidences of PONV when the Consensus Guidelines are Followed vs. the Proposed Perphenazine-Dexamethasone-Ondansetron Technique**

| Baseline PONV Risk | Consensus-Recommended Intervention | Consensus-Dosed, PONV Cases per 200 | P-D-O Technique, PONV Cases per 200 | PONV Prevented with P-D-O, Cases per 200 |
|---|---|---|---|---|
| 10% | Wait and see | 20 | 8 | 12 |
| 20% | Wait and see | 40 | 16 | 24 |
| 40% | One or two antiemetics | 53 | 32 | 21 |
| 60% | Two or three antiemetics | 57 | 48 | 9 |
| 80% | Two or three antiemetics | 76 | 64 | 12 |
| ***Difference in incidence (column total)*** | | ***246 per 1000*** | ***168 per 1000*** | ***78 cases per 1000 prevented*** |

P-D-O Technique: perphenazine (8 mg orally before surgery) plus dexamethasone (4 mg i.v. after induction) plus ondansetron (4 mg i.v. before emergence)

When the consensus-recommended intervention involves a range of options (e.g., one to two antiemetics, or two to three antiemetics), the PONV cases per 200 represents a weighted average.

In this illustration, oral perphenazine is assumed to be therapeutic equivalent to intravenous droperidol. Further prospective study (beyond that described in Desilva et al.[3]) is needed to confirm this clinical impression.

No other multimodal techniques are assumed to have been given in either treatment arm (e.g., regional or total intravenous anesthesia).

Based on this estimate, 78 fewer patients per 1000 would encounter PONV with the P-D-O technique, representing a 32% risk reduction when compared with the consensus guideline.

## SUMMARY

For 10 years, we have used intravenous and oral perphenazine as part of a multimodal antiemetic prophylaxis care plan in at least 10,000 outpatients. We have never encountered an adverse event, to our knowledge, when the intravenous dose was less than or equal to 2 mg, or when the single preoperative oral dose did not exceed 8 mg (with no repeated dosing). As a single-dose component of multimodal antiemetic prophylaxis therapy, we believe that this track record of anecdotal safety in adults who meet certain criteria (age 14–70, no less than 45 kg, no history of extrapyramidal reactions or of Parkinson disease, and no Class III antidysrhythmic being coadministered for coexisting disease) constitutes a sufficient patient safety basis for formal prospective study. We believe that future perphenazine studies should include routine coadministration with prospectively established multimodal antiemetics (i.e., dexamethasone and a 5-$HT_3$ antagonist). Oral perphenazine 8 mg should be rigorously evaluated in patients receiving coadministered dexamethasone and 5-$HT_3$ antagonists in order to determine differences in synergistic efficacy, if any. Similar trials should be performed, individually evaluating cyclizine, transdermal scopolamine, and aprepitant in combination with coadministered dexamethasone and a 5-$HT_3$ antagonist. Such studies should also quantify efficacy in preventing nausea and vomiting after discharge home, and also quantify the extent to which the prophylaxis plans reduce PACU requirements (i.e., increase PACU bypass), reduce the need for any nursing interventions for PONV, and influence the extent to which any variable costs of postoperative nursing care are reduced.
